# New SAMD9L heterozygous mutation leading to myelodysplastic syndrome and acute myeloid leukemia: A case report and review of the literature

**DOI:** 10.1002/cnr2.1797

**Published:** 2023-03-07

**Authors:** Dania A. Monagel

**Affiliations:** ^1^ College of Medicine King Saud bin Abdulaziz University for Health Sciences Jeddah Saudi Arabia; ^2^ King Abdullah International Medical Research Center Jeddah Saudi Arabia; ^3^ Department of Oncology Ministry of the National Guard‐ Health Affairs Jeddah Saudi Arabia

**Keywords:** acute myeloid leukemia, myelodysplastic syndrome, pediatric, SAMD9L, transplant

## Abstract

**Background:**

SAMD9L mutation is linked to the development of myeloid neoplasm. The mutation has a wide range of clinical presentations involving neurological, immunological, and hematological manifestations. Until now, limited data regarding different variants of this genetic mutation existed. Here we present a 6‐year‐old girl who presented with acute myeloid leukemia/myelodysplastic changes and who carries a new germline variant mutation in the SAMD9L gene.

**Case Presentation:**

A 6‐year‐old girl who presented initially as a case of immune thrombocytopenic purpura (ITP) was later diagnosed with acute myeloid leukemia and myelodysplastic changes. In addition, she was found to have a new germline variant mutation in the SAMD9L gene (other known pathogenic variants known to cause ataxia pancytopenia syndrome). She was treated with chemotherapy followed by haplo identical transplant from her unaffected father. She is alive 30 months post‐transplant and in complete remission with full donor chimerism. Her initial brain MRI showed mild prominence of the anterior (superior) vermis folia, suggesting mild atrophy. Ongoing surveillance for accompanied neurological manifestation is ongoing, although the patient is asymptomatic.

**Conclusion:**

For SAMD‐9L‐related disorder, a careful approach must be taken when a patient presents with a suspicious clinical feature even without a well‐known genetic mutation giving the diverse presentation across affected members within the same family. In addition, other associated abnormalities should be monitored long‐term.

## 1 INTRODUCTION

Familial bone marrow failure (BMF) syndromes usually present during childhood. Few germline mutations in genes like RUNX1, DDX41, ETV6, ANKRD26, GATA2, SAMD9, SAMD9L have been associated with the pathogenesis of inherited myelodysplastic syndromes (MDSs).^1^ Here, we report a 6‐year‐old girl with MDS/ acute myeloid leukemia (AML) with a new variant in the SAMD9L gene that was treated successfully with haploidentical hematopoietic cell transplantation (HCT).

## 2 CASE REPORT

A 6‐year‐old girl was admitted to our hospital (King Abdul‐Aziz Medical City‐ Jeddah) as a case of pancytopenia for investigations. She had a history of isolated thrombocytopenia for 3 years prior to her presentation. She was treated as a chronic immune thrombocytopenic purpura with intravenous immunoglobulin (IVIG) and observation. She had a febrile illness for 3 weeks before her admission, and her complete blood count (CBC) showed pancytopenia with 7% peripheral blast. Otherwise, her past medical history was unremarkable. She has a 10‐year‐old sister who is healthy and has no history of consanguinity between her parents. Her family history was insignificant for cancers, inherited bone marrow failure, or MDS. Her physical exam was unremarkable for any dysmorphic feature, telangiectasias, and she had a normal neurological examination. Her lab work showed normal immunoglobulins (9.92 g/L, reference range 5.40–13.60 g/L) and alpha‐fetoprotein levels (<2 ng/mL, reference range 1–4 ng/mL).

### 2.1 Initial bone marrow assessment

Bone marrow aspirate showed active erythropoiesis with megaloblastic changes and dysplastic nucleus. Granulopoiesis was reduced, left‐shifted, and dysplastic. Megakaryocytes were reduced and dysplastic. Bone marrow revealed the presence of storage iron and the absence of ringed sideroblast. Other findings include a blast of approximately 22%, low N/C ratio, some show fine granulation, Auer rods. Bone marrow trephine displayed around (60%–70%) cellularity, interstitial infiltration by the blast, reduced dysplastic megakaryocytes, and normal reticulin fibers. The findings were consistent with AML with myelodysplastic‐related changes.

**FIGURE 1:**
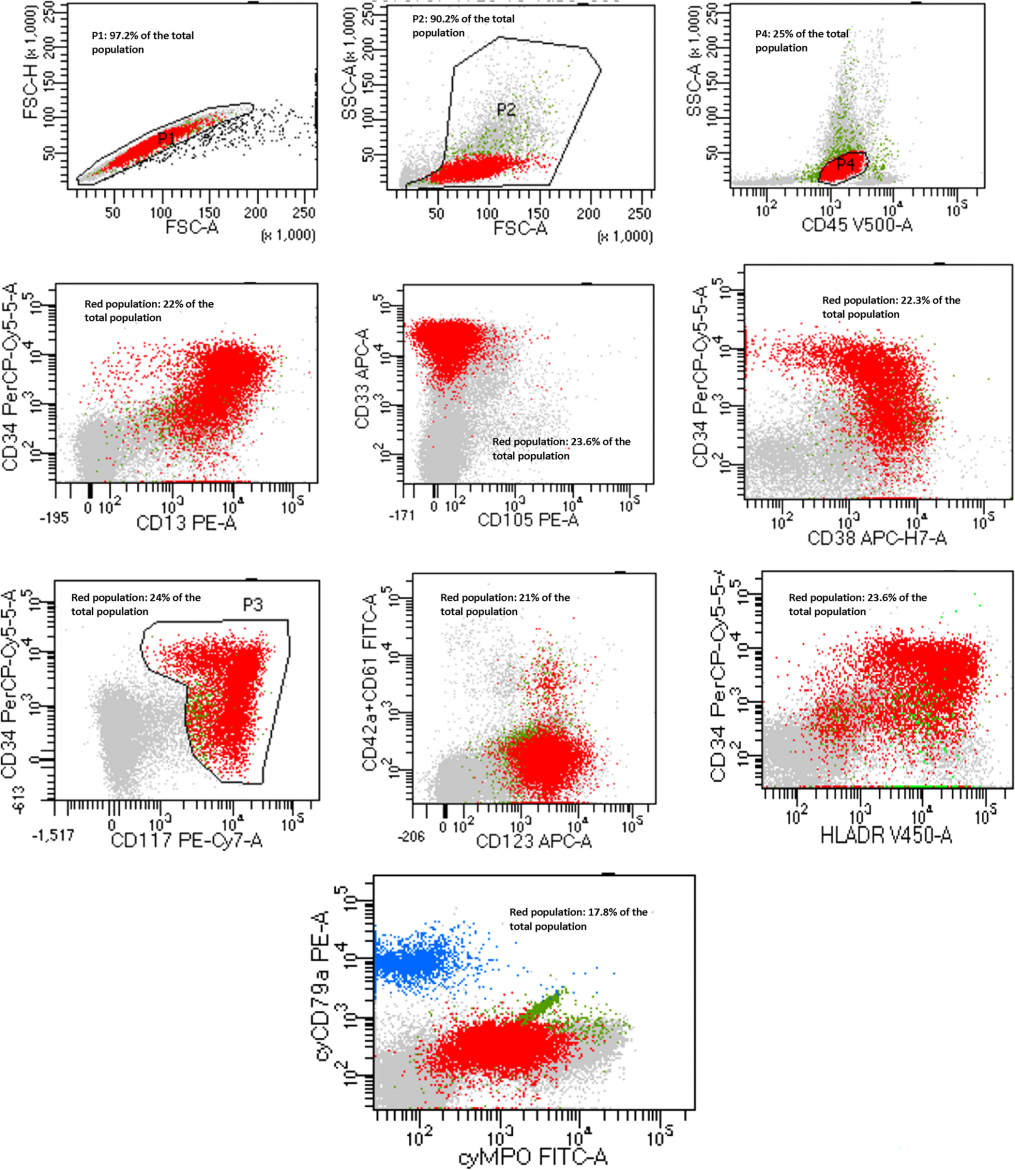
Dot‐plot histograms from flow cytometric analysis of the bone marrow aspirate from our patient show SSC/CD45 dim population as the blast (25% cell population in blast region). Blasts (red populations) show positivity for CD13, CD33, CD34, CD38, CD117, CD123, & HLADR with partial expression of CD34; dim expression of CD4 and MPO; partial/dim expression of CD7, CD15 and CD19. The flowcytometric analysis used BD FACSDiva™ Software. CD45 gating was applied (CD45‐SSC). Events ranged from 30 000 to 50 000 events; percentages of each population are displayed for selected population of interest in each plot in relation to total population (Total population defined as total numbers of events in the tube of bone marrow sample).

Flow cytometric analysis of bone marrow sample showed around 25% cell population in the blast region (CD45:dim) expressing CD13, CD33, CD34, CD38, CD117, CD123, & HLADR with partial expression of CD34; dim expression of CD4 and MPO; partial/dim expression of CD7, CD15 and CD19 (Figure 1). Negative for CD2, s&cyCD3, CD10, CD11b, CD14, CD16, CD22, CD25, CD35, CD36, CD41, CD42, CD56, CD61, CD64, cyCD79a & TDT.

Chromosome analysis revealed an abnormal female chromosome complement with the gain of chromosome 11 as the sole anomaly in 18 out of all cells examined. The remaining two cells showed a normal female chromosome complement. Trisomy 11 as the sole abnormality is a recurrent, non‐random abnormality seen primarily in acute myeloid leukemia and myelodysplasia and is generally associated with an intermediate to poor prognosis.

Karyotype (ISCN 2016): 47, XX, +11 [18]/46, XX [2].

Next genome sequence analysis revealed the mutation c.2039C > T in the FLT3 gene.

She was started on our local protocol for the treatment of AML and received two cycles of ADE (ARA‐C 100 mg/m^2^/dose IV every 12 h for 20 doses cycle 1 and 16 doses cycle 2, daunorubicin 50 mg/m^2^/dose IV on Days 1–3‐5, and etoposide 100 mg/m^2^/dose IV daily on Days1‐5). She was in remission post ADE1. However, her bone marrow showed slow recovery post ADE1, especially for platelets, which never recovered (50–70 × 10^9^/L).

**FIGURE 2:**
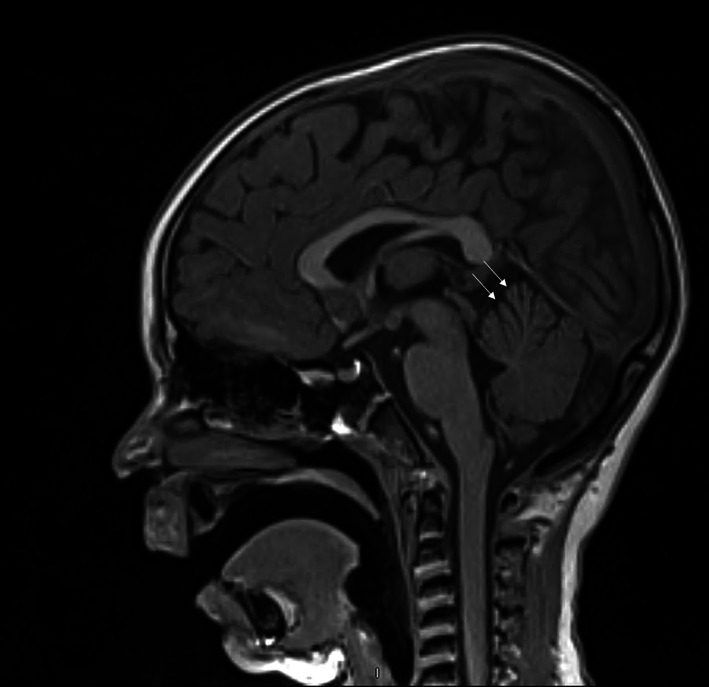
Sagittal T1 brain MRI shows mild atrophy of the anterior lobe of the cerebellum.

She has a 10/10 matched sibling, but due to serious concern of underlying inherited BMF/MDS, we sent a whole‐exome sequence (WES) analysis. Her WES came back +ve for SAMD9L heterozygous mutation of unknown significance (based on ACMG recommendation). The variant was c.1949A > G p.(Glu650Gly) chr7:92763336. In the literature, the pathogenic variants in the SAMD9L gene lead to ataxia pancytopenia syndrome with a predisposition to AML, MDS, and bone marrow failure, which is present in this case. A segregation study for the family indicated that both her mother and sister have the same mutation but are asymptomatic with normal CBC. A magnetic resonance image (MRI) brain was performed even without neurological manifestations and showed mild prominence of the anterior (superior) vermis folia, suggesting mild atrophy with no other brain abnormalities (Figure 2).

In the absence of enough evidence to guide our decision, our transplant team decided to go ahead with haploidentical HCT using her father as a donor over the use of her fully matched sister, who carries the same mutation. She underwent a haploidentical transplant with post‐transplant cyclophosphamide (50 mg/kg/day on Day +3 and Day +4) and cyclosporin (2.5 mg/kg/dose IV q 12 h, target level 200–250 ng/mL) for graft versus host disease (GVHD) prophylaxis. The conditioning regimen consisted of thiotepa (10 mg/kg/dose on Day −7), melphalan (140 mg/m^2^/dose on day −6), and fludarabine (40 mg/m^2^/dose on day‐5 till day −2). The choice of conditioning was based on limited resources during the peak of the COVID‐19 pandemic (in particular, the access to total body irradiation and busulfan therapeutic drug monitoring). Therefore, peripheral blood was used as a stem cell source. She developed stage 2, grade 1 skin graft versus host disease treated with topical steroids. She remained on cyclosporine for 6 months following her HCT with the successful withdrawal of all her immune suppression. She has 100% donor chimerism and is currently alive, in remission, with complete normalization of her CBC 30 months post HCT.

## 3 DISCUSSION

Here we described a whole‐exome sequencing (WES) that revealed not previously reported variant c.1949A > G p.(Glu650Gly) in the SAMD9L gene. The patient presented with a clinical manifestation that fit the picture of ataxia pancytopenia syndrome (ATXPC). She was treated successfully with HCT using a haploidentical transplant approach to treat her AML.

Sterile α motif domain‐containing protein 9 (SAMD9) and SAMD9‐like (SAMD9L) are interferon‐inducible antiviral restriction factors, and their role in bone marrow failure or neurological defects is poorly understood.^2^, ^3^ It is located on chromosome 7q21.3 in the region recurrently deleted in myeloid neoplasm.^4^ The mutations in those genes can lead to rare disorders of unknown prevalence, which have an autosomal dominant mode of inheritance. MIRAGE syndrome (myelodysplasia, infection, restriction of growth, adrenal hypoplasia, genital phenotypes, and enteropathy) has been linked to gain of‐ function in the SAMD9 gene. In contrast, different disease entities have been related to the mutation in SAMD9L, including ataxia pancytopenia syndrome (ATXPC), isolated MDS, and most recently, SAMD9L‐associated autoinflammatory disease (SAAD).^5^, ^6^

Table 1 summarized the cases with different variants in the SAMD9L gene reported to date that had been associated with variable disease phenotypes even within the same family. Comparing our patients with what had been reported in the literature, our case showed a full clinical picture of the hematological manifestation of ATXPC but only radiological evidence of neurological presentations. She harbors a unique mutation that was not linked before to this illness. Long‐term follow‐up is required for the patient and her family as her onset of the ataxia or acquiring the disease by another family member may become evident over time. The use of haplo transplant approach was successful in her case and allows further insight into such modality for this kind of mutation in the future.

**TABLE 1:** Literature review of SAMD9L cases.

Disease phenotype	Number of patients	Patients demographic	Clinical feature	SAMD9L variants	Treatment	Outcome	Reference
SAAD	2 subjects	1 Male neonate/Chinese 1 Female neonate /Korean	Fever, Hepatosplenomegaly, Skin rash, Epiglottitis, Perforated bowel, Calcification of basal ganglia, Dysplasia of bone marrow, Low platelet, Anemia, Low or absent B cell	‐chr7‐92 762 631‐T‐; NM_152703.4:c.2654delA; (p.Asn885Thrfs*6) ‐chr7‐92 762 652‐T‐; NM_152703.4: c.2633delA; (p.Lys878Serfs*13)	‐Steroid, MMF ‐ One patient improved by 4 years of age. ‐ BMT for the second patient	‐ 1 alive at 11y ‐ 1 Died during BMT	5
SAAD	6 subjects	1 male, other NS	Nodular panniculitis and lipoatrophy, interstitial lung disease and basal ganglia calcifications	‐c.2666delT; p.F889Sfs*2 ‐c.2658_2659delTT/ F886Lfs*11 ‐c.2626delA;p. I876Lfs*15 ‐c.2633delA; p.K878fs*13	Steroid, IVIG, BMT, Tocilizumab, Anakinra, Adalimumab, Etanercept	−2 alive post BMT −2 died (no BMT) −2 alive (no BMT)	11
ATXPC	12 subjects (2 families)	‐Age (5 m to 61 y) Family1:Swedish Family2:Finnish	Cytopenia, MDS, Recurrent infection, Nystagmus, Ataxia, ADHD	‐c.2956C.T, p.Arg986Cys ‐c.2672 T.C, p.Ile891Thr mutations	2 had BMT for MDS	−9/10 alive ‐Some had spontaneous resolution	9, 12
ATXPC	6 subjects (same family)	−5 male, 1 female ‐Started from infancy ‐Polish	‐All have ataxia ‐ Cytopenia ‐ AML	No genetic test	Steroid /androgen	4/6 died	13, 14
ATXPC	7 subjects (same family)	‐ Age (12 m to 5y) −5 male /2 female ‐Northern European family	‐ALL, ataxia, cytopenia, macrocytosis, AA	‐(c.4418G > A, NM_152703.4) encoding a p.Ser1473Asn (S1473N) missense substitution.	‐BMT in ALL patient ‐Some had resolution AA: oxymetholone	2/7 died	10
ATXPC	1 subject	‐Age 12y MDS ,28 y onset of neuro symptom ‐Female	‐ataxia and MDS	(NM_152703.5:c.2956C > T, p.(Arg986Cys))	NS	Alive	15
ATXPC	15 subjects, (2 families)	‐2Female/7 male ‐ rest NS ‐ Irish, German, & Native American	F1: ataxia/ variable cytopenia F2: ataxia, cytopenia/ AML	‐c.2640C > A ‐c.3587G > C,p.Cys1196Ser	Supportive care	−3/9 died −5/6 died	16
ATXPC	3 subjects (1 family)	−2 female, 1 male	‐Onset of ataxia early within 5y of life −1 mild thrombocytopenia −1 AML −1 normal CBC	‐Not done, monosomy 7	AML therapy	1/3 died AML	17
ATXPC	10 subjects	‐Age: 8 m – 46y	BMF, ataxia, nystagmus, transient cytopenia	‐c.G3442C p.D1148H ‐c.A4477G p.I1493V ‐c.T4648C p.S1550P ‐c.2852_2853insACAC p.T951fs ‐c.T2519A p.M840K ‐c.C4561G p.L1521V ‐c.A3427G p.S1143G ‐c.C2956T p.R986C (*n* = 2) ‐c.G3100T p.D1034Y	6/10 had BMT	1/10 died	8
ATXPC	2subject (Same family)	1Female, at 3 m Father (healthy)	Leukoencephalopathy, demyelinating peripheral neuropathy, Dural ectasia, Pancytopenia	‐c.2686 T > G, p.(Phe896Val)	Supportive, cytopenia resolved	alive	18
Familial thrombocytopenia MDS	16 subjects	‐7female, 9 male ‐ Age: 9 m‐ 37y	Thrombocytopenia, 8 MDS, 1 AML, 3 AA/PNH	Variants: Trp517Arg, I327V, L1323fs, L50S, P123L, E220G, G235S, W333C, A637T, S728P, Y705C, R1298G, A637S, G247A	‐supportive for thrombocytopenia, others: NS	NS	1
MDS AML, transient monosomy 7	14 subjects (4 families)	NS	AML, MDS, transient monosomy 7	c.C2640A p.H880Q c.C2956T p.R986C c.T4535C p.V1512A c.G3842A p.R1281K	NS	NS	19
MDS/AML	6 subjects (4 families)	−3 male, 3 Female Age: 7 m‐ 12 y	MDS, AML, 1 had additional HLH	‐c.1877C > T; p.S626L ‐c.3538 T > C; p.W1180R ‐c.4651 G > C; p.V1551L ‐c.2957G > A; p.R986H	‐All had BMT −1 graft failure	2 died, (1 refractory AML, 1 VOD)	4
MDS	7 subjects (4 families)	‐Age (1y to 42y) −5 male/2 female ‐German/ Swedish	All had thrombocytopenia with/without additional cytopenia and a hypocellular marrow without an increase of blasts, monosomy 7 ‐No Dysmorphic features or neurological symptoms	‐p.H880Q ‐p.R986H ‐p.R986C ‐p.V1512M	5/7 had BMT	−6/7 alive, 1 died post BMT ‐ 2 recovered spontaneously ‐ 2 progress to leukemia	6
MDS	4 subjects	−3 female, 1 male ‐ Age (1y‐12y)	MDS	‐p.W1180R ‐p.S626L (*n* = 2) ‐p.R1281K	NS	1 died	20

Abbreviations: AA, aplastic anemia; ALL, acute lymphoblastic leukemia; AML, acute myeloid leukemia; ATXPC, ataxia pancytopenia syndrome; BMF, bone marrow failure; BMT, bone marrow transplantation; F1, family 1; F2, family 2; MDS, myelodysplastic syndrome; MMF, mycophenolate; NS, not specified; PNH, paroxysmal nocturnal hemoglobinuria; SAAD, SAMD9L‐associated autoinflammatory disease.

Cerebellar ataxia, cytopenia, progressive bone marrow failure, myelodysplasia (MDS), and predisposition to myeloid malignancy with or without monosomy 7 are characteristic features of ATXPC.^2^ The disorder has a different age of onset of hematological/neurological manifestations with intrafamilial phenotypic variability.^2^ Even patient who has cerebellar atrophy on the brain MRI may remain asymptomatic, while others may have mild hematological involvement. Pastor et al. reported 30% of the mutation carrier to be asymptomatic in their series.^6^ Heterozygosity of the SAMD9L gene of uncertain significance does not establish or rule out the disease, and the clinician should maintain a high index of suspicion and correlate that with the clinical course of a given individual.^2^ Interestingly, cis heterozygous loss of those genes on the same chromosome leads to myelodysplastic syndrome, while an individual may tolerate a biallelic loss of one gene.^7^

Ahmed et al. had the largest series on transplant outcomes for children with germline SAMD9/SAMD9L mutations. They reported successful resolution of MDS or bone marrow failure, with a high survival rate.^4^ Six patients were included in their cohort, and 11 had been reported in the literature with SAMD9L mutations, underwent transplantation for MDS with chromosome 7 abnormalities, and received myeloablative or reduced‐intensity conditioning with stem cells mostly from unrelated donor grafts. Eight subjects are alive post HCT; two died and 1 with unknown status. The majority had no neurological issues before HCT.^4^, ^6^, ^8^, ^9^ Time and variable indications for HCT in SAMD9L patients are not identified as some may go through the spontaneous recovery of their blood count.^4^, ^10^

Hence, we recommend that although HCT is the only curative approach to the hematological aspect of the disease, some patients can still be managed with supportive care measures, especially if they do not show any sign of AML/MDS. A high index of suspicion for this genetic mutation should be kept in mind when dealing with children with MDS or other disease‐related manifestations, giving the significant heterogeneity even within the same pedigree. Any potential related donor should be screened for the mutation as we did in our case. In addition, continuous surveillance of other disease‐related manifestations should be carried out even post HCT.

## 4 CONCLUSION

This case illustrates the current established clinical feature of a form of SAMD9L genetic mutation, which can cause ataxia pancytopenia syndrome. This variant was not described previously but highlights that additional knowledge of this entity will allow easier detection in the future.

## AUTHOR CONTRIBUTIONS

**Dania Monagel:** Conceptualization (lead); data curation (lead); formal analysis (lead); methodology (lead); project administration (lead); resources (lead); software (lead); writing – original draft (lead); writing – review and editing (lead).

## CONFLICT OF INTEREST STATEMENT

The authors have stated explicitly that there are no conflicts of interest in connection with this article.

## ETHICS STATEMENT

This manuscript was approved by our institutional ethics board. Informed consent was taken from the patient's legal guardian.

## Data Availability

The data that support the findings of this study are available on request from the corresponding author. The data are not publicly available due to privacy or ethical restrictions.
